# Acceptance and adoption determinants of telemedicine in public healthcare institutions

**DOI:** 10.4102/curationis.v48i1.2604

**Published:** 2025-01-16

**Authors:** Kokisa E. Phorah, Lovemore Motsi

**Affiliations:** 1 School of Computing, College of Science, Engineering and Technology, University of South Africa, Florida, South Africa; 2 School of Computing, College of Science, Engineering and Technology, University of South Africa, Pretoria, South Africa

**Keywords:** information and communication technology (ICT), North West hospitals, technology acceptance model (TAM), telemedicine, user acceptance

## Abstract

**Background:**

One of the challenges facing the usage of telemedicine technology in South Africa, particularly in the North West province (NWP), is lack of user acceptance by health care professionals which prevents piloted project to produce the desired outcomes.

**Objectives:**

The purpose of this study was to investigate the influential factors for the adoption of telemedicine by health care professionals from three selected hospitals (Bophelong, Taung and Klerksdorp) in the NWP.

**Method:**

The study adopted a case study approach and gathered data through questionnaires, which were distributed to the health care professionals of the three selected hospitals. In order to ensure that the instrument was accurate, a pilot study was carried out prior to the main investigation. Both the alpha and power values were set at 0.05 for the statistical analysis. The statistical tool used for the data analysis was SPSS v. 23.0.

**Results:**

Attitude towards the use of telemedicine technology (ATUTT), perceived usefulness (PU) and acceptance of telemedicine technology (ATT) were considered to be the influential factors in the adoption of telemedicine technology. The association between perceived ease of use (PEOU) and perceived usefulness (PU) (0.150, *p* = 0.034), PEOU and Attitude towards the use of telemedicine technology (ATUTT) (0.211, *p* = 0.002), PEOU and Acceptance of telemedicine technology (ATT) (0.245, *p* = 0.000), PU and (ATT) (0.212, *p* = 0.002), ATUTT and (ATT) (0.189, *p* = 0.005). However, PEOU was found to have an insignificant relationship with (0.048, *p* = 0.093). Hypotheses 1, 3, 4, 5, and 6 are supported while H2 was not supported.

**Conclusion:**

The study aims to fill the knowledge vacuum identified, helping poor countries effectively implement telemedicine technology to modernise the health care sector. In addition, results from this study shed insight on the varied impacts of individual, technical, clinical and multidimensional social influence variables on health care providers’ decisions to use telemedicine.

**Contribution:**

This study contributes to the body of knowledge by investigating the success factors for telemedicine technology adoption in South African public hospitals. These results have important implications for the public health care system in South Africa, both for the direction of future research and for the methods used to promote the use of telemedicine.

## Introduction

The health care industry is one of the many industries that can benefit from the competitive advantage that technology offers in terms of process improvement and strategic realisation (Alolayyan et al. 2020; Chen, Lin & Wu 2020). Furthermore, the health care industry has undergone a rapid transformation, with manual operational systems being replaced by digital health care technologies such as personal health records, electronic prescriptions, smart health devices, wearable technologies, artificial intelligence (AI)-enabled patient relationship management and telemedicine. These technologies have had a significant impact on the industry. Health care professionals and their patients face serious challenges in adopting these technologies. (Chau et al. 2019; Popov et al. 2022; Thapa et al. 2021). A new functional systems design (Kaium et al. 2020) or a favourable shift in the public health care sector (Dash 2020) could result from utilising cutting-edge technology like the Internet of Things (IoT) and AI in the health and medical professions. The term ‘telemedicine’ was used by Bashshur et al. (2020) to describe a patient-centred method of distant health care delivery that makes use of digital technologies. Dick et al. (2020) stated that mobile health services have been more widely available because of technological advancements in the past decade.

Telemedicine enables health care professionals to provide care from their homes and has proven to be a valuable tool to decrease the crowded waiting rooms (Mann et al. 2020). Other reported benefits were the probability of having a work–life balance, patient access and providing care to more patients than before, along with simplicity and user-friendliness (Bunnell et al. 2020). Telemedicine is considered timesaving and reduces the costs associated with travelling (Chu et al. 2020). Sclafani et al. (2021) reported shorter appointments with telemedicine. Nearly all participants reported that the consultations were less than 30 min, and half of the participants reported that consultations were less than 15 min. Nearly half of the participants in the study by Svider et al. (2020) reported that they spent between 5 and 15 min on appointments. Gold et al. (2021) found that the majority thought it was easy to use video consultations and would use it again, and they agreed that it was less time-consuming than regular face-to-face consultations. The benefits of telemedicine are many; cost-effective, such as reduced costs and low expenses is one of them, and timesaving, such as decreased travel time (Bunnell et al. 2020; Mackowiak 2021; Mgbako et al. 2020).

### Background

According to Abdullah, Jafta and Chapanduka (2020), due to the growing demand for healthcare resources and the necessity of optimising hospital operations, it is imperative to provide healthcare services that are efficient. In this situation, it is imperative to resolve urgent concerns while simultaneously motivating healthcare professionals to innovate and enhance services. In order to ensure that patients receive accurate results from laboratory testing and diagnostic and physical examinations, the authors have demonstrated that health care facilities must have a suitable number of competent health workers. Telemedicine is currently indispensable and is anticipated to endure. It allows healthcare professionals to broaden the scope of their direct and urgent medical support services. It facilitates healthcare accessibility in any location that has a communications network (Paczka-Giorgi et al. 2021). Furthermore, telemedicine is expected to remain a significant factor in the delivery of healthcare, as it is capable of accommodating patient preferences and requirements in a manner that in-person clinical consultations are unable to meet (Paczka-Giorgi et al. 2021). Unfortunately, the healthcare system in the North West province is confronted with a variety of obstacles, including lengthy wait times, shortage of medical staff, and the inability to access medical facilities (Ndinda et al. 2018). Some of the issue is due to the fact that the majority of medical professionals in the public sector are not employed in rural areas, which are home to half of the nation’s population. In addition, the difficulties of providing high-quality health care in this politically charged region of the province have received surprisingly little attention from researchers.

Telemedicine has developed into an essential aspect of the South African Department of Health’s e-health initiative, with more than R15 million funded in various telemedicine projects in recent years (Motsoaledi 2010). Despite these investments, it is only believed that 34% of telemedicine sites are operational, which shows that the technology is still in its infancy (Motsoaledi 2010; Van Dyk, Fortuin & Schutte 2012). The low adoption of telemedicine is not unique to South Africa, as several authors have noted equivalent results in other developing countries (Gajarawala & Pelkowski 2021; Ovretveit et al. 2007). Previous studies found that the system’s reliability was compromised by frequent power outages, poor connectivity and constrained capacity (Kwangsawad & Jattamart 2022; Shankar & Nigam 2022). Since telemedicine’s introduction, these problems have been rectified (Burgener 2020), but adoption has remained modest (Battineni et al. 2021). As a result, a brand-new area of study is now required, with a focus on the organisational and interpersonal problems (Caffaro et al. 2020; Kemp et al. 2021). One of the factors that has been identified as a potential roadblock to the successful implementation of telemedicine is user acceptance (Alsabeeha et al. 2022; Lin et al. 2023). There is a rising failure rate in many organisations, including the health care sector, as a result of the absence of readiness evaluations undertaken prior to the deployment of new systems or policies (Garavand et al. 2022).

However, telemedicine brings a blanket of hope to underprivileged rural remote areas. In general, these locations remain underserved as they lack the presence of working professionals (Shahpori et al. 2018). In addition to its primary function of increasing accessibility to remote health services, telehealth can help address gaps in access to healthcare services and health outcomes as a result of the “brain drain” of medical healthcare professionals who are leaving the country for more promising opportunities (Shahpori et al. 2018). Piloted telemedicine projects were implemented and evaluated by a few hospitals in South Africa’s North West province beginning in 1999 (Gulube & Wynchank 2002). The hospitals are Bophelong Provincial Hospital, Taung District Hospital and Klerksdorp-Tshepong Hospital, functioning as sending sites and receiving sites. These piloted public healthcare facilities have not been able to function as telemedicine facilities in South Africa because of the numerous challenges the country faces.

The inability of the government to provide the populace with high-quality health care services has also been linked to poor health care infrastructure and a large population (Edward et al. 2015). Prior to the implementation of any new systems, it is crucial to evaluate the organisational and health personnel readiness in the health care sector. This is mostly because evaluating an organisation’s preparedness requires establishing if all of its resources, including its facilities and infrastructure, are ready to embrace the new system (Ross, Ressia & Sander 2017).

This study was conducted in a few hospitals in the North West province of South Africa. The hospitals selected for this study were Bophelong Provincial Hospital, Taung District Hospital and Klerksdorp-Tshepong Hospital. Because of their participation in the initial telemedicine trial study, these hospitals were selected. These hospitals serve as district and provincial hospitals in South Africa. The technology acceptance model (TAM) was chosen as the primary theoretical framework for the investigation.

## Literature review and hypotheses

The TAM is acknowledged as the most significant model used to determine the elements influencing the adoption of information technology (IT) in the health care system as well as patients’ attitudes and behaviours when it comes to health care technology, according to Garavand et al. (2017). According to Harst, Lantzsch and Scheibe (2019), TAM is the most prevalent tool for assessing telemedicine adoption by measuring the intention to use the technology. In this study, perceived usefulness (PU), perceived ease of use (PEOU), behavioural intention (BI) and actual usage constructs were adopted from TAM.

**FIGURE 1:**
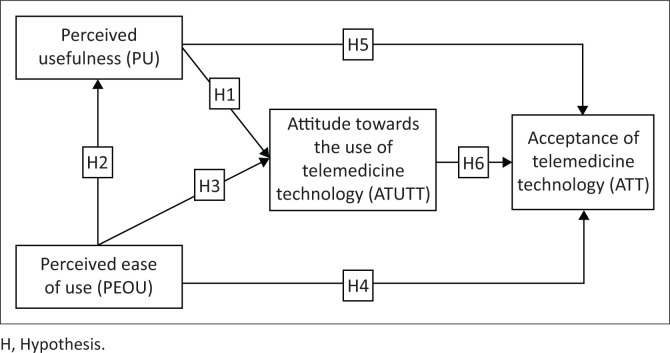
Conceptual framework including hypotheses.

Despite the fact that the original TAM had significant limitations, Davis (1989) recommended modifying it to include additional variables dependent on the circumstances of the study. In order to expand TAM to include relevant variables and how they affect telemedicine acceptance, research is necessary. To suit the objectives of this study, attitude towards use was modified to read health care professionals’ attitude towards the use of telemedicine technology (HATT) and was changed to read health care professionals’ acceptance of telemedicine technology (UATT). The conceptual framework used in this study is illustrated in Figure 1.

### Hypotheses development

According to Caffaro et al. (2020) and Wu and Chen (2017), PU refers to how much a person thinks adopting a particular technology will enhance their ability to accomplish their work. The study’s concept of PEOU states that telehealth technology will increase the effectiveness of health care practitioners. Numerous studies (Venkatesh & Morris 2000; Wamba et al. 2017) have supported the effect of PU on ATUTT, particularly in the context of people’s openness to adopting new technologies like smartphones and the IoT (Kabbiri et al. 2017). The idea of usefulness is somewhat vague when it comes to health-related themes like e-health (Venugopala, Jinkab & Priyac 2016), telehealth (Gajarawala & Pelkowski 2021; Tsai et al. 2019) or telemedicine solutions (Sun et al. 2013). The following are the corresponding hypothesises:

**H1:** Perceived usefulness has a direct positive influence on the attitude towards the use of telemedicine technology.

**H5:** Perceived usefulness has a direct positive influence on the attitude towards the use of telemedicine technology.

According to Al-Rahmi et al. (2019) and Binyamin (2020) PEOU is the degree to which a person thinks utilising a certain technology will be simple. Numerous studies using the TAM approach are supported by the idea that product uptake and usage are linked to perceptions of usefulness (Guner & Acarturk 2020). This demonstrates that clients will adopt new technology when they think it is obviously useful or advantageous. Perceived ease of use may have an impact on PU. Perceived ease of use has been researched by several researchers in the context of the adoption of new technologies in the health care industry (Baudier, Ammi & Lecouteux 2019; Abd-alrazaq et al. 2019). According to Gajarawala and Pelkowski (2021), a higher PEOU for the telemedicine service may indicate that its users find it to be of more value. The corresponding hypotheses are as follows:

**H2:** Perceived ease of use has a direct positive influence on the ease of use of telemedicine technology.

**H3:** Perceived ease of use has a direct positive influence on the attitude towards the use of telemedicine technology.

**H4:** Perceived ease of use has a direct positive influence on the attitude towards the use of telemedicine technology.

Related research (AlQudah et al. 2021; Albarrak et al. 2021) revealed that health care professionals had favourable attitudes towards ICT. Studies showing that views towards ICT are frequently favourable, including those by Kunwar et al. (2022) and Fouad et al. (2023), provide additional support for the linked studies. Because attitudes have a significant influence on behaviour, the effectiveness of implementing innovative health care technology rests in great part on the attitudes of health care professionals and service users (Kruse et al. 2018). Usage intention is a variable commonly used to analyse the factors influencing the acceptance of information technology (Yang et al. 2021). The corresponding hypothesis is as follows:

**H6:** Attitude towards the use of telemedicine technology has a direct positive influence on technology acceptance.

## Research methods and design

Health care professionals in South Africa’s North West province were the study population of a cross-sectional quantitative study that aimed to gauge their level of preparedness. Cross-sectional studies, as stated by Schmidt and Brown (2019), are conducted by taking a moment in time and analysing it in order to ascertain the frequency of a particular event that occurs within a society. In agreement with Schmidt and Brown (2019), the researcher will hand out a questionnaire to a subset of respondents and then return it to them once they have finished filling it out. In scientific studies, the word ‘population’ is used to refer to the entire group of study population, objects or occurrences that are being examined because of a common characteristic. It includes everything the researcher plans to look at and draw conclusions from, says Ngulube (2022). Following the utilisation of the G*power software to ascertain the minimum sample size of 89 required for this study, the populations were chosen through the utilisation of simple random sampling.

Participation was open to medical health care professionals: obstetricians, nutritionists, psychiatrists, optometrists, pharmacists, occupational therapists, dentists or psychologists working at three public hospitals (Klerksdorp, Taung and Mahikeng). To collect this information, we used a questionnaire. In order to ensure that the instrument was accurate, a pilot study using SPSS Statistics for Windows, Version 23.0 (IBM SPSS Statistics for Windows, Armonk, New York, US). Both the alpha and power values were set at 0.05 for the statistical analysis. A self-administered questionnaire with open and closed-ended questions was developed for the study. The questionnaire comprised 15 questions that were designed to assess health care professionals PU, PEOU and ATUTT. The questionnaire was developed using the conceptual framework shown in Figure 1 as a guide. Responses ranged from ‘strongly disagree’ to ‘strongly agree’, with scores ranging from 1 to 5.

### Statistical data analysis

The questionnaires were then coded and transcribed into the SPSS v 23.0 for analysis. Frequency tables were used for the descriptive analysis. Associations among variables were seen using correlation analysis and hypothesis testing to investigate the influential factors for the adoption of telemedicine by health care professionals from three selected hospitals (Bophelong Provincial Hospital, Taung District Hospital and Klerksdorp-Tshepong Hospital) in the North West province.

### Ethical considerations

To conduct an ethical study, researchers must follow certain rules. Ethics-related issues serve as principles and directives for conducting ethically sound research. Such ethical procedures are intended to preserve the security of personal information of all participants, thus fulfilling the guarantee made to them under the informed consent form. The use of an informed consent form also helped ensure that participants were fully informed of their rights and protections and that they had provided full consent to participate in the study. Ethical approval to conduct this study was obtained from the University of South Africa, College of Science, Engineering and Technology Ethics Sub-Committee (reference no.: 024/KEP/2015).

## Results

### Demographic characteristics of respondents

Of the 120 questionnaires that were distributed, 98 were completed, rendering a response rate of 84.2% which was considered acceptable for analysis. The majority of participants were nurses (75.5%), followed by doctors (7.1%), radiologists (4.1%) and pharmacy staff (24.9%) as shown in Table 1. This is in line with the actual distribution of health care providers in South Africa, as well as in other countries which usually employ more nurses than physicians and other health providers. With respect to gender, the male staff (50.5%) and female staff (49.5%) were relatively equally distributed, and the results also show that the higher group of respondents (64.4%) belonged to the younger age population – under the age of 30 years. Moreover, the placements of the participants were equally distributed between the hospitals. Regarding the level of education, 62.4% of the sample possessed a secondary qualification, 18.8% had a university diploma, while the rest possessed either a degree or postgraduate degree qualification. It is also important to point out that many of the participants (66.3%) had less than 10 years of experience.

**TABLE 1:** Demographic characteristics of respondents (*N* = 98).

Demographic Variable	Frequency	Sample (%)
**Gender**		
Male	56	57.14
Female	42	42.85
**Age group (years)**		
30–39	28	28.57
40–49	34	34.69
50–59	21	21.43
> 60	15	15.31
**Level of experience**		
< 10	57	58.16
< 20	25	25.51
21–30	17	17.34
> 30	3	3.06
**Level of education**		
Certificate	19	19.39
Diploma	52	53.06
Bachelors’ degree	17	17.35
Masters’ degree	4	4.08
Other	8	8.16
**Occupation**		
Nurse	74	75.51
Doctor	7	7.14
Pharmacist	3	3.06
Dentist	2	2.04
Psychiatrist	2	2.04
Dietician	3	3.06
Psychologist	3	3.06
Radiologist	4	4.08

### Correlation analysis

The correlation analysis conducted in this study provides valuable insights into the relationships between PU, PEOU, ATUTT and acceptance of telemedicine technology (ATT) among the selected hospitals in the North West province. The Pearson’s correlation coefficients reveal varying degrees of correlation between these variables, with all *p*-values being less than 0.05, indicating statistically significant relationships.

### Strength of the correlations

1. *PU and ATUTT*: The correlation coefficient of *r* = 0.604 indicates a strong positive correlation between PU and ATUTT. This suggests that as individuals perceive telemedicine technology to be more useful, their attitude towards its adoption becomes increasingly favourable. This robust relationship implies that enhancing the perceived benefits of telemedicine could significantly improve attitudes towards its use, leading to higher acceptance rates in health care settings.
2. *PEOU and PU*: The correlation coefficient of *r* = 0.338 shows a moderate positive correlation between PEOU and PU. This indicates that when telemedicine is perceived as easy to use, it tends to be viewed as more useful as well. The practical implication of this finding is that simplifying the user experience of telemedicine applications can enhance their perceived value, encouraging health care professionals and patients to adopt these technologies more readily.
3. *ATUTT and ATT*: The correlation coefficient of *r* = 0.289 suggests a weak but positive relationship between ATUTT and ATT. While this indicates that a more favourable attitude towards telemedicine does contribute to acceptance, the weaker correlation suggests that other factors may also play a significant role in determining acceptance levels. Therefore, while improving attitudes is essential, it may not be the sole strategy for increasing telemedicine adoption; a broader approach that addresses additional factors influencing acceptance is warranted.
4. *PEOU and ATUTT*: The correlation coefficient of *r* = 0.279 indicates a weak positive correlation between PEOU and ATUTT. This relationship suggests that a more favourable perception of the ease of using telemedicine technologies contributes to a positive attitude towards their adoption. Practically, this means that simplifying telemedicine systems and providing effective training can help foster more positive attitudes, which may, in turn, influence acceptance.
5. *PU and ATT*: The correlation coefficient of *r* = 0.642 indicates a strong positive relationship between PU and ATT. This relationship implies that when health care professionals recognise the usefulness of telemedicine technologies, they are more likely to accept and use them. This finding underscores the importance of demonstrating the practical benefits of telemedicine, such as improved patient outcomes and increased efficiency, to encourage widespread adoption.

### Directionality of relationships

The positive directionality of the relationships found in this analysis suggests that as one variable increases, the others also tend to increase. For instance, the positive influence of PEOU on ATUTT indicates that when users find telemedicine applications easy to navigate and use, they develop a more favourable attitude towards these technologies. This relationship is crucial in practice; health care organisations should focus on enhancing user experiences through intuitive design, comprehensive training and ongoing support to foster positive attitudes towards telemedicine.

Furthermore, the positive correlation between PU and ATT indicates that perceptions of the usefulness of telemedicine are directly linked to the willingness to accept and utilise these technologies. Health care stakeholders must prioritise communication strategies that highlight the tangible benefits of telemedicine to enhance acceptance among users. By addressing both PU and PEOU, health care providers can effectively influence attitudes towards telemedicine adoption, thereby supporting broader implementation efforts in the health care system.

### Hypothesis testing

In this section, a summary of the research findings is provided in relation to the six hypotheses of this study. Table 2 shows the summary result of the hypothesis testing.

**TABLE 2:** Hypothesis testing.

Hypotheses	Coefficient	*t*-value	*p*	Recommendation
H1	0.150	2.014	0.034	Supported
H2	0.045	1.689	0.093	Not supported
H3	0.211	3.104	0.002	Supported
H4	0.245	3.245	0.000	Supported
H5	0.212	3.122	0.002	Supported
H6	0.189	2.148	0.005	Supported

Five out of the six hypothesised relationships are statistically significant at the 0.05 level or higher. The results of the hypothesis testing reveal that H1, H3, H4, H5, and H6 are supported. Specifically, H1 (0.150, *p* = 0.034), H3 (0.211, *p* = 0.002), H4 (0.245, *p* = 0.000), H5 (0.212, *p* = 0.002), and H6 (0.189, *p* = 0.005) all show positive coefficients and statistically significant *p*-values (below 0.05), indicating meaningful relationships between the variables. These supported hypotheses suggest that factors such as infrastructure, support, and system quality are significantly related to the outcomes being studied. Conversely, H2 (0.048, *p* = 0.093) is not supported, as its *p*-value exceeds the significance threshold of 0.05, suggesting that the hypothesized relationship does not hold statistically. These findings highlight the importance of focusing on specific quality factors in improving outcomes.

## Discussion

One of the six hypotheses that were examined in this study was rejected, and the hypotheses were based on the TAM (Davis 1989).

**H1:** PU having a direct impact on ATUTT was accepted based on the findings of correlation analysis and hypothesis testing. The great majority of research using the TAM approach includes the assumption that user acceptance and the use of new technology are correlated with their feelings of usefulness (Caffaro et al. 2020). This suggests that users will accept new technology when they perceive its utility or benefit as being unmistakably advantageous. The TAM makes the assumption that a person’s impression of how simple a technology is to use influences their assessment of its usefulness (Davis 1989). The legitimacy of TAM is further supported and validated by this study, which also highlights the importance of two antecedents in the original TAM for attitudes towards use and perceived utility of digital health technology. The results of this study are in line with those of earlier studies (Faqih & Jaradat 2021; Al-Maroof et al. 2021; Yang et al. 2016) when it comes to the importance of PU and attitude towards use.

**H2:** PEOU has a positive significant influence on PU was rejected. The results are in contradiction to those of earlier researchers. Not all scholars arrived at the same result from the perspective of the two key TAM model constructs, PEOU and PU. According to the study by Singh, Sharma and Paliwal (2020), PEOU had no negative effects on the attitudes of users or interfered with PU. The hypothesis that suggested a positive significant between the PEOU and the PU was not validated, according to Vladova et al. (2021). Regarding the relationship between PEOU and PU and attitude, Vladova et al.’s (2021) study reached the same conclusion. Further research is necessary to understand the reasons why this hypothesis was rejected because of the inconsistent relationship between PEOU and PU. However, as demonstrated in Table 3, there was a significant correlation between PEOU and ATUTT in terms of the use of telemedicine technology.

**TABLE 3:** Correlation matrix.

Variable	PU	PEOU	ATUTT	ATT
**PU**				
Pearson’s correlation	1.00	-	-	-
Sig. (2-tailed)	-	-	-	-
**PEOU**				
Pearson’s correlation	0.34**	1.00	-	-
Sig. (2-tailed)	0.00	-	-	-
**ATUTT**				
Pearson’s correlation	0.60**	0.28**	1.00	-
Sig. (2-tailed)	0.00	0.00		-
**ATT**				
Pearson’s correlation	0.21*	0.74**	0.29**	1.00
Sig. (2-tailed)	0.03	0.00	0.00	-

PU, perceived usefulness; PEOU, perceived ease of use; ATUTT, attitude towards the use of telemedicine technology; ATT, acceptance of telemedicine technology; Sig., significance.

* , Correlation is significant at the 0.05 level (2-tailed).

** , Correlation is significant at the 0.01 level (2-tailed).

**H3:** PEOU has a positive significant influence on ATUTT was accepted. This is in line with research findings from Intention to use an information system is influenced by TAM’s underlying theory in addition to PU and simplicity of use. However, it is still consistent with findings from earlier studies that show attitude and PU have an impact on the intention to use social networking tools (Andavara et al. 2021). As demonstrated in Table 3, there was a favourable correlation between PEOU and ATUTT and the adoption of telemedicine technology. This implies that the attitude towards using telemedicine technology will increase in proportion to how useful it is seen to be.

**H4:** PEOU has a positive significant influence on ATUTT was accepted. This finding is consistent with other research findings, which indicate that PEOU has a substantial impact on intentions. As a result, difficult electronic procedures should be avoided to encourage users to use electronic system-based technologies. This result suggests that users will value a system more and vice versa depending on how simple they find it to use. Attitudes towards the use of telemedicine technology and PEOU have been correlated in similar ways in earlier investigations. According to Table 3, there is a significant positive association between PEOU and ATUTT with regard to the adoption of telemedicine technology.

**H5:** PU has a direct positive influence on the attitude towards the use of telemedicine technology was accepted. The results of the current investigation showed that PU had a significant positive influence on ATT. This implies that PU, which was also perceptible in earlier research, is a factor affecting ATT. This outcome is consistent with research by Ghaddar et al. (2020), who found that attitudes influence inclinations to use telehealth services favourably. Therefore, this fits with one’s attitude towards the technology that will be used affecting their intention to use it (Gajarawala & Pelkowski 2021). As demonstrated in Table 3, there was a positive significant correlation between PU and ATT and the adoption of telemedicine technology.

**H6:** ATUTT has a positive significant influence on ATT was accepted. As shown in Table 3, there is a positive significance correlation ATUTT and ATT. This is consistent with the findings of earlier studies. According to Ajzen and Schmidt (2020), attitudes towards behaviour are a function of beliefs that may result in actions that produce particular outcomes or particular experiences. In addition, PU and convenience of use have an impact on usage intentions (Prakosa & Sumantika 2020). Therefore, several studies (Bakshi & Tandon 2022; Lee et al. 2019) focussed on behavioural intention to utilise technology to predict the actual use and technological acceptability.

### Critical reflection on study limitations

Despite the insightful findings, several limitations need to be acknowledged. Firstly, the study primarily relied on cross-sectional data, which captures a snapshot in time and does not account for changes in attitudes and perceptions over time. Longitudinal studies would provide a richer understanding of how acceptance of telemedicine evolves as users gain more experience with the technology. Additionally, the use of self-reported measures could lead to response biases, where participants may overstate their positive attitudes or underestimate challenges associated with telemedicine. Incorporating objective measures, such as usage statistics or performance metrics, could enhance the robustness of the findings.

Furthermore, the study’s sample was limited to health care professionals in the North West province, potentially limiting the generalisability of the results to other regions or health care settings. Variability in local health care systems, cultural attitudes towards technology and access to resources could influence telemedicine adoption differently in diverse contexts. Expanding the sample to include a wider range of health care professionals across various regions would yield more comprehensive insights into the factors affecting telemedicine acceptance. Lastly, the exclusion of other potentially influential variables – such as organisational culture, infrastructure readiness and training – could provide a more nuanced understanding of telemedicine adoption. Future research should aim to include these variables to develop a holistic model that captures the complexity of telemedicine acceptance in health care settings.

## Conclusion and further research

This study investigated the factors influencing the adoption of telemedicine at three hospitals in North West province. The study showed that PU has a significant positive influence on ATUTT, PEOU has a significant positive influence on ATUTT, PU has a large positive influence on ATT, and ATUTT has a significant positive influence on ATT. Compared to PU and PEOU, ATUTT and ATT were thought to be the strongest predictors of telemedicine acceptance. Testing revealed a positive correlation between the relationships between telemedicine technology and PU and ATUTT, PU and ATT, ATUT and ATT.

The findings of this study showed that PEOU had a negative insignificant influence on PU and was rejected. The findings conflict with those of earlier researchers. From the standpoint of the two important TAM constructs, PEOU and PU, not all researchers reached the same conclusion. However, it was discovered that there was a positive significant correlation between PEOU and PU. Although the response rate was high, there were some restrictions in this study, such as the small sample size. The respondents could perhaps not fully comprehend the driving forces behind telemedicine adoption. Future researchers are advised to take into account additional factors, such as age-related characteristics, physical changes, digital literacy and adoption in order to present a wider perspective and more conclusive view on the behavioural intention of the elderly health care professional to use digital health care wearables in order to provide a deeper understanding of this study.
